# Moderating Effects of Physical Activity and Global Self-Worth on Internalizing Problems in School-Aged Children With Developmental Coordination Disorder

**DOI:** 10.3389/fpsyg.2018.01740

**Published:** 2018-09-19

**Authors:** Yao-Chuen Li, Jeffrey D. Graham, John Cairney

**Affiliations:** ^1^Institute of Population Health Sciences, National Health Research Institutes, Miaoli, Taiwan; ^2^INfant and Child Health (INCH) Lab, Department of Family Medicine, McMaster University, Hamilton, ON, Canada; ^3^Faculty of Kinesiology and Physical Education, University of Toronto, Toronto, ON, Canada

**Keywords:** mental health, environmental stress hypothesis, motor coordination, buffering effect, moderation

## Abstract

School-aged children with developmental coordination disorder (DCD) are at greater risk for physical inactivity, lower global self-worth, and internalizing problems, such as depression and anxiety. Based on the environmental stress hypothesis (ESH), recent research has shown that physical inactivity and lower global self-worth sequentially mediate the relationship between DCD and internalizing problems, suggesting that DCD leads to lower levels of physical activity, which in turn leads to lower levels of global self-worth, and ultimately, a greater amount of internalizing problems. However, physical activity and global self-worth may also buffer (i.e., moderate) the adverse effect of DCD on internalizing problems. To date, this has yet to be tested. Participants were 1206 children aged 12–14 years [611 boys, 79 with probable DCD (pDCD)]. All children received assessments of motor coordination, physical activity, global self-worth, and internalizing problems. Children with pDCD were less physically active, had lower self-worth, and experienced more internalizing problems compared to typically developing (TD) children (*p*’s < 0.05). Furthermore, the moderated moderating effect (three-way interaction) of physical activity and global self-worth was also evident (*p* < 0.05), indicating that internalizing problems in both TD and pDCD groups decreased with concurrent increases in physical activity and global self-worth. Importantly, when compared to TD children, increases in physical activity and global self-worth were associated with a greater reduction in internalizing problems among children with pDCD. The findings support several pathways in the ESH and highlight that, in addition to improving motor skills, interventions should also target both physical activity and global self-worth to mitigate potential mental health issues for children with motor difficulties.

## Introduction

A growing number of studies have been conducted to investigate physical and mental health in children with developmental coordination disorder (DCD; Mancini et al., 2016a). DCD is one of the most common neurodevelopmental disorders of childhood, affecting about 2–6% of all school-aged children. It is characterized by severe motor impairments that neither result from neurological/musculoskeletal diseases, nor visual or intellectual disabilities (American Psychiatric Association [APA], 2013). However, the motor impairment characteristic of DCD leads to a variety of difficulties including limitations in activities of daily living at home (e.g., typing shoelaces or zipping up a jacket) and performing academic tasks at school (e.g., handwriting or using scissors), as well as limitations in the participation of physical activities that require motor coordination or skills (e.g., riding a bike or dribbling a ball; American Psychiatric Association [APA], 2013). In addition to difficulties in motor tasks and limitations in participation, children and adolescents with DCD have consistently been found to have poor psychosocial well-being, such as lower levels of self-efficacy, global self-worth, and perceived social support (Cairney et al., 2005; Piek et al., 2008; Mancini et al., 2016b).

Recent studies have consistently found that children, adolescents, and adults with DCD (or poor motor coordination) are at a greater risk for internalizing problems, such as anxiety and depression, compared to their typically developing (TD) peers (Piek et al., 2007; Pratt and Hill, 2011; Campbell et al., 2012; Lingam et al., 2012; Hill and Brown, 2013; Wilson et al., 2013; Missiuna et al., 2014; Mancini et al., 2016b; Rigoli et al., 2017). Current evidence suggests that poor mental health may track from childhood into emerging adulthood in this population as poor motor coordination identified at early ages is associated with emotional problems and depressive symptoms later in life, specifically in girls (Wagner et al., 2016; Harrowell et al., 2017). Furthermore, as DCD is highly comorbid with attention deficit/hyperactive disorder (ADHD), school-aged children with both DCD and ADHD have been reported by parents to have higher levels of depression, compared to those with either DCD only or ADHD only, or without any disorder (Piek et al., 2007; Missiuna et al., 2014). Collectively, these findings highlight the importance of both early identification and intervention related to mental health problems in children with DCD.

It is worth noting, in spite of the fact that emerging research has investigated mental health problems in children with DCD, the underlying mechanisms of internalizing problems in children with DCD have not been systematically discussed until recent years. Gaining a greater understanding of the underlying mechanisms is important to aid in the design of effective interventions. To date, the environmental stress hypothesis (ESH) has been the only model that was specifically developed to explain higher levels of internalizing problems in children with DCD (Cairney et al., 2013). According to the ESH, children with DCD experience more internalizing problems due to a cascade of adverse effects of DCD on physical health (i.e., physical activity and weight status) and psychosocial well-being (i.e., general stress, perceived social support, and self-concept). In brief, DCD, conceptualized in the ESH as a primary stressor, increases the risk of a number of risk factors for poor mental health, including physical inactivity and overweight/obesity, which in turn deteriorate a child’s psychosocial well-being (e.g., sense of self; perceived efficacy), and ultimately, results in a greater risk for internalizing problems (Cairney et al., 2013; Mancini et al., 2016a).

Since the ESH was first proposed, a burgeoning set of studies have been conducted examining several of the mediating pathways outlined in the ESH across a number of different age groups. For example, Wilson et al. (2013) found that social skills mediated the relationship between motor ability and emotional symptoms in children aged 4–6 years. Mancini et al. (2016a) found a mediating effect of perceived family support on the relationship between motor skills and depressive symptoms among 12- to 16-year-old children; a finding which has also been replicated in adults aged between 18 and 30 years (Wilson et al., 2013; Mancini et al., 2016b; Rigoli et al., 2017). Interestingly, recent research has also found evidence for a sequential mediation pathway (Li et al., 2018), proposed in the ESH, which suggests a more complicated mechanistic pathway leading from DCD to internalizing problems that cannot be investigated using single mediation analyses. Specifically, Li et al. (2018) found that in both school-aged boys and girls, DCD led to lower levels of physical activity, which then led to lower levels of global self-worth and, in turn, higher levels of internalizing problems. However, an important hypothesis outlined in the ESH, the potential buffering (or protecting) effect of these mediating variables on internalizing problems, has not been examined thus far.

Based on the literature reviewed above, some constructs outlined in the ESH, specifically physical activity, perceived social support, and positive self-concept, may serve as moderators that could buffer the negative impact of DCD on mental health (Cairney et al., 2013). In other words, although children with DCD may have more internalizing problems compared to TD children, these consequences may be mitigated if they have higher levels of physical activity, perceive more support from parents or friends, and/or have a more positive self-concept, such as higher levels of self-esteem, mastery, and self-competence. These moderating effects are particularly interesting as DCD is a heterogeneous group, and not every child with DCD may show the same characteristics (i.e., be less physically active, or perceive lower levels of social support and self-concept). Therefore, examination of potential moderating effects will not only enhance our understanding of the underlying mechanisms of internalizing problems in children with DCD, but may also help health professionals develop more effective interventions targeting mental health in this population.

**FIGURE 1:**
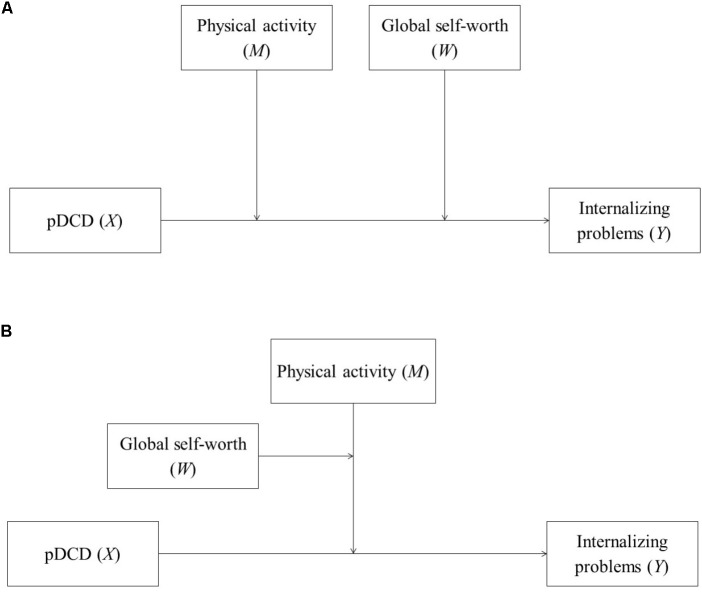
**(A)** Additive moderation model of physical activity and global self-worth. **(B)** Moderated moderation model of physical activity and global self-worth. pDCD, probable developmental coordination disorder.

The overarching purpose of this study was to test multiple moderation models to understand the potential buffering effect(s) of physical activity and global self-worth (represented as self-concept in this study) on the relationship between DCD and internalizing problems based on the ESH (Cairney et al., 2013). As a previous study has shown that the children with DCD have lower levels of physical activity and self-worth as well as more internalizing problems when compared to their TD peers (Li et al., 2018), we hypothesized (H1) that physical activity and global self-worth would each (separately) moderate the relationship between DCD and internalizing problems. However, based on specific pathways outlined in the ESH, we sought to investigate whether these two variables may also interact with one another in a way that may further explain the relationship between DCD and internalizing problems. Specifically, two advanced moderation models were tested. First, as suggested by Cairney et al. (2013), both physical activity and global self-worth are hypothesized to moderate the relationship between DCD and internalizing problems. Therefore, an *additive moderation* model was tested to examine the collective (or additive) moderating effects of physical activity and global self-worth on the relationship between DCD and internalizing problems (as depicted in **Figure 1A**). We hypothesized (H2) that higher levels of physical activity and global self-worth would buffer (i.e., weaken) the adverse effect of DCD on internalizing problems (i.e., significant two-way interaction of DCD by physical activity and DCD by global self-worth on internalizing problems).

As previously mentioned, recent research has also shown that the relationship between DCD and internalizing problems is indirectly affected by the effects of physical activity on global self-worth (Li et al., 2018). For instance, Li et al. (2018) found that DCD led to lower levels of physical activity, which then led to lower levels of global self-worth and, in turn, higher levels of internalizing problems. These findings highlight the unique, and sequential, pathway whereby DCD affects internalizing problems through the changes in physical activity on global self-worth. However, they also suggest that these two variables may interact with one another in way that could buffer (i.e., weaken) the negative effects of DCD on internalizing problems. Therefore, a second model examined the moderating effect of physical activity on global self-worth and, ultimately, this interaction effect on the DCD-internalizing problems relationship (i.e., *moderated moderation*; see **Figure 1B**). Specifically, unlike the additive moderation model where physical activity and global self-worth could simultaneously, yet separately, moderate the DCD-internalizing problems relationship, moderated moderation takes into account how one moderator could affect another (also see Hayes, 2013, pp. 300–315). Thus, based on the sequential mediating pathways discussed above, it was hypothesized (H3) that physical activity (i.e., primary moderator) would interact with global self-worth (i.e., secondary moderator), whereby higher levels of physical activity would serve as a protective mechanism on levels of global self-worth which, collectively, would lead to fewer internalizing problems resulting from DCD (i.e., significant three-way interaction of DCD by physical activity by global self-worth on internalizing problems).

## Materials and Methods

### Participants and Procedure

This study was a cross-sectional analysis using the eighth wave of the Physical Health and Activity Study Team (PHAST) project, a large prospective cohort study investigating children’s physical and psychosocial health. Data collection was conducted in the southern region of Ontario, Canada, between October and December 2008. The sample included 1206 children (611 boys, 50.7%) between the ages of 12 and 14 years (*M*age = 13.4 ± 0.3 years, 12.4–14.8 years) with valid data on motor coordination, physical activity, global self-worth, and internalizing problems. Of these, 79 children (6.6% of the sample, 30 boys) who scored at or below the 10th percentile on the Bruininks–Oseretsky Test of Motor Proficiency – Short Form (BOTMP-SF) were identified as probable DCD (pDCD). Wave 8 was the first and only wave to include a measure of psychological distress and this is the reason we are only using one wave of data from this study.

A detailed description of the study procedure is provided elsewhere (Cairney et al., 2009). In brief, children’s motor skills and height/weight were assessed in a school gymnasium by trained research assistants. Children were also asked to complete questionnaires assessing levels of physical activity, global self-worth, and internalizing problems. Informed, written consent was provided by parents. Ethical approval was obtained from the local District School Board and Brock University.

### Measures

#### Internalizing Problems – Kessler-6 (K6) scale

The Kessler-6 (K6) scale was developed to measure psychological distress, which is conceptually similar to internalizing problems (Kessler et al., 2002). It consists of six items that ask the frequency (1 = none of the time, 2 = a little of the time, 3 = some of the time, 4 = most of the time, 5 = all of the time) of different feelings in the past 30 days, including feeling nervous, hopeless, restless or fidgety, depressed, and worthless as well as perceptions of tasks feeling effortful. Item scores are summed to a total score ranging from 0 to 24; higher scores indicate a higher level of internalizing problems. A large number of subjects had missing data on this variable (*n* = 442, 33.4%), so a multiple imputations method was used to deal with missing values. This procedure is described in greater detail elsewhere (Li et al., 2018). The K6 scale has demonstrated excellent internal consistency (Cronbach’s α = 0.89–0.92) and discriminative validity, indicating that it is able to differentiate individuals with and without mental disorders (Kessler et al., 2002). The K6 scale showed good internal consistency in this sample α = 0.82.

#### Motor Coordination – Bruininks–Oseretsky Test of Motor Proficiency – Short Form (BOTMP-SF)

The BOTMP is a validated standardized test (Bruininks, 1978) that is commonly used to evaluate children’s motor skills and identify motor impairments (Crawford et al., 2001; Cairney et al., 2010). The short version of the BOTMP (i.e., BOTMP-SF), which contains 14 items, was used in this study to assess fine motor control, manual coordination, body coordination, and strength/agility. The BOTMP-SF has been shown to have excellent agreement with the long version of the BOTMP (*r* = 0.90–0.91; Bruininks, 1978). Importantly, the BOTMP-SF has been validated as an appropriate assessment tool for the diagnosis of children with DCD when compared with the more widely used Movement Assessment Battery for Children (M-ABC; Henderson and Sugden, 1992; also see Cairney et al., 2009).

Raw scores for each item were converted into point scores that were further summed and converted into standard scores based on age- and sex-matched norms. Percentile ranks were also converted to identify children with pDCD (i.e., at or below the 10th percentile). Taking into account the relative stability (*r* = 0.70, *p* < 0.001) of motor abilities in children during this developmental stage (at least in the absence of interventions), the motor assessment was only conducted once for most participants in this study (Cairney et al., 2009). The use of the 10th percentile was to produce a threshold that was more in-line with estimated prevalence of DCD (American Psychiatric Association [APA], 2013) and has been used before in previous research (Cairney et al., 2005, 2007; Li et al., 2018). In fact, the recommended threshold for caseness on the BOTMP is above the 10th percentile, but this can lead to miss-classification. The fact that the 10th percentile threshold identified about 6% of children brings our sample estimate in line with known prevalence.

#### Physical Activity – Participation Questionnaire (PQ)

The Participation Questionnaire (PQ) is a 63-item self-report questionnaire measuring children’s participation in different domains of physical activity, such as free play, team sports, or dance clubs (Hay, 1992). Children’s responses to each item were scored to create activity units that were then summed as a count of activities. Subscale scores were calculated for free play and organized activity, both of which were further summed to generate a total score with a higher total score indicating a higher level of participation in physical activity. The PQ has been validated to show high test–retest reliability (r = 0.81–0.89) as well as construct validity showing significant sex and urban/rural differences in activity levels (Hay, 1992).

#### Global Self-Worth – Harter’s Self-Perception Profile for Children (SPPC)

The Self-Perception Profile for Children (SPPC) measures children’s perceptions of self-competence, such as athletic, social, and academic competence as well as overall perceptions of self-worth (Harter, 1985). Construct and convergence validity of the SPPC have been validated, and good internal consistency has been previously found (α = 0.78–0.87; Harter, 1985, 2012), as well as in this study (α = 0.86). The global self-worth subscale from the SPPC was used in the present study to represent “personal resources” outlined in the ESH. The subscale consists of six items regarding children’s perceptions about themselves and their lives. Children were first asked to read two opposite statements for each question (e.g., “some kids are happy with themselves as a person” or “other kids are often not happy with themselves”); then, they needed to indicate the statement that best applied to them alongside the strength of the statement (i.e., “sort of true for me” or “really true for me”). A total score of the six items was used to represent global self-worth, with higher scores indicating greater perceptions of self-worth.

### Statistical Analysis

Data on all children were included in each analysis. Descriptive statistics were conducted using SPSS 22 for Windows (Armonk, NY, United States: IBM Corp.). Independent sample *t*-tests and chi-square tests were used to examine group differences.

Tests of moderation were conducted using the PROCESS software macro (Hayes, 2013). First, two simple moderation models were examined. In these models, the moderating effect of physical activity and global self-worth were separately tested on the relationship between pDCD and internalizing problems (H1). To do so, PROCESS estimated the main effects of pDCD and the moderator (i.e., physical activity *or* global self-worth) and then computed the two-way interactions (i.e., pDCD by physical activity *or* pDCD by global self-worth) on the relationship between pDCD and internalizing problems (default Model 1 in PROCESS). A significant two-way interaction would indicate significant simple moderation.

Two advanced moderation models were then examined. In order to test H2 (as shown in **Figure 1A**), an additive moderation of physical activity (*M*) and global self-worth (*W*) was examined on the relationship between pDCD/TD (*X*) and internalizing problems (*Y*) as suggested by the ESH (Cairney et al., 2013). The main effects of pDCD and the moderators (i.e., physical activity *and* global self-worth) were estimated, and then two two-way interactions (i.e., pDCD by physical activity *and* pDCD by global self-worth) on the relationship between pDCD and internalizing problems were computed (default Model 2 in PROCESS). Significant two-way interactions would indicate significant additive moderation.

In order to test H3 (**Figure 1B**), a moderated moderation model was tested for physical activity (*M*) and global self-worth (*W*) based on previous research (Li et al., 2018). Similar to the examination of H2, main effects and two-way interactions were computed. Furthermore, a three-way interaction (i.e., pDCD by physical activity by global self-worth; default Model 3 in PROCESS) was also computed. Taking into account the pathways observed in previous research (Li et al., 2018), physical activity was considered as the primary moderator, whereas global self-worth was the secondary moderator. A significant three-way interaction would indicate significant moderated moderation.

It is worth noting that sex has been previously identified to be an important factor influencing physical activity, global self-worth, and internalizing problems in school-aged children. For example, compared to boys, girls have been found to be less physically active (Cairney et al., 2010), have lower levels of self-concept (Schmidt et al., 2015), and report more internalizing problems (Wade et al., 2002). Moreover, a previous study examining the mediating pathways from pDCD to internalizing problems in the ESH found that the underlying mechanisms through physical activity and global self-worth may also be sex specific. For instance, the single mediation effects of physical activity and global self-worth were only found in girls, whereas the sequential mediation of physical activity and global self-worth explained the relationship between pDCD and internalizing problems in both boys and girls (Li et al., 2018). Thus, sex was controlled for in all tested models in the present study. The statistical significance for all tests was set at α < 0.05.

## Results

### Between Group Comparisons

Descriptive statistics for the study measures are shown, by group, in **Table 1**. There were no significant differences in age (*t* = -1.32, *df* = 1204, *p* = 0.19) and height (*t* = 0.42, *df* = 1204, *p* = 0.68) between children with and without pDCD. There were significantly more boys in the TD group than the pDCD group (*x*^2^ = 5.445, *df* = 1, *p* < 0.05). When compared to TD children, school-aged children with pDCD had higher weight and BMI scores, more internalizing problems, poorer motor coordination, lower levels of physical activity, and lower global self-worth (all *p*’s < 0.05; medium to large effect size).

**Table 1:** Descriptive statistics of children with and without pDCD (*M* ± SD or %).

	TD (*n* = 1127)	pDCD (*n* = 79)	*t-*statistic	*p-*value	Cohen’s *d*
Boys (%)	581 (51.6%)	30 (38.0%)	5.445^a^	0.020	0.067^b^
Age (years)	13.40 ± 0.33	13.45 ± 0.38	–1.316	0.189	
Height (cm)	160.75 ± 7.75	160.37 ± 8.07	0.418	0.676	0.048
Weight (kg)	54.14 ± 12.32	64.66 ± 17.87	–5.150	<0.001	0.685
BMI (kg/m^2^)	20.84 ± 3.93	24.90 ± 5.49	–6.467	<0.001	0.850
Internalizing problems	5.48 ± 4.14	7.80 ± 6.34	–3.210	0.002	0.433
BOTMP-SF (%ile score)	73.27 ± 24.73	4.29 ± 0.20	84.123	<0.001	3.912
Physical activity	14.13 ± 5.88	10.11 ± 4.44	7.608	<0.001	0.772
Global self-worth	20.23 ± 3.42	18.83 ± 4.61	2.324	0.020	0.345


*^a^Pearson chi-square statistic was reported; ^b^Cramer’s V was used to indicate the effect size. TD, typically developing; pDCD, probable developmental coordination disorder; BMI, body mass index; BOTMP-SF, Bruininks–Oseretsky Test of Motor Proficiency – Short Form. Table revised from Li et al. (2018).*

### Moderation Analyses

#### H1: Simple Moderation of Physical Activity and Global Self-Worth

There was a significant direct effect of pDCD on internalizing problems (unstandardized coefficient = 2.206, SE = 0.501, *p* < 0.001).ffects for pDCD on internalizing problems (both *p*’s < 0.05) were found in both models, the interaction terms of pDCD by physical activity (coefficient = -0.130, SE = 0.111, *p* > 0.05) *or* pDCD by global self-worth (coefficient = -0.216, SE = 0.117, *p* > 0.05) on internalizing problems were not significant.

#### H2: Additive Moderation of Physical Activity and Global Self-Worth

As shown in **Table 2**, additive moderation model explained 13.7% of variance in internalizing problems. While controlling for sex (coefficient = 0.728, SE = 0.235, *p* < 0.01), there were significant main effects of both physical activity (coefficient = -0.059, SE = 0.021, *p* < 0.01) and global self-worth (coefficient = -0.336, SE = 0.030, *p* < 0.001) on internalizing problems. However, there were no significant two-way interactions of pDCD by physical activity (coefficient = -0.090, SE = 0.107, *p* > 0.05) and pDCD by global self-worth (coefficient = -0.205, SE = 0.117, *p* > 0.05) on internalizing problems in this model.

**Table 2:** The effects of additive moderation and moderated moderation on the relationship between DCD and internalizing problems.

	Simple moderation		Additive moderation	Moderated moderation
	Coefficients (SE)	Coefficients (SE)	Coefficients (SE)	Coefficients (SE)
Intercept	3.047 (1.377)*	5.615 (2.294)*	5.234 (0.167)***	5.184 (0.167)***
**Main effect**				
pDCD	3.200 (1.246)*	5.576 (2.070)**	1.118 (0.615)†	0.949 (0.618)
PA	0.048 (0.117)		–0.059 (0.021)**	–0.064 (0.021)**
GSW		–0.117 (0.128)	–0.336 (0.030)***	–0.316 (0.031)***
**Two-way interaction**				
pDCD^∗^PA	–0.130 (0.111)		–0.090 (0.107)	–0.119 (0.107)
pDCD^∗^GSW		–0.216 (0.117)†	–0.205 (0.117)†	0.107 (0.166)
PA^∗^GSW				0.013 (0.005)*
**Three-way interaction**				
pDCD^∗^PA^∗^GSW				0.052 (0.024)*
**Covariate**				
Sex	0.783 (0.247)**	0.781 (0.235)***	0.728 (0.235)**	0.750 (0.235)**
R^2^	0.042^∗∗∗^	0.131^∗∗∗^	0.137^∗∗∗^	0.145^∗∗∗^


*^†^p < 0.10, ^∗^p < 0.05, ^∗∗^p < 0.01, ^∗∗∗^p < 0.001. pDCD, probable developmental coordination disorder; PA, physical activity; GSW, global self-worth.*

#### H3: Moderated Moderation of Physical Activity and Global Self-Worth

When the moderated moderation model was tested, the three-way interaction term was the main interest of this study among all predictors, and their effects on internalizing problems are reported. As seen in **Table 2** (moderated moderation model), when sex was adjusted (coefficient = 0.750, SE = 0.145, *p* < 0.01), there was a significant three-way interaction of pDCD by physical activity by global self-worth (coefficient = 0.052, SE = 0.024, *p* < 0.05), indicating a significant moderated moderation of physical activity and global self-worth on the relationship between pDCD and internalizing problems. The model accounted for 14.5% of variance in internalizing problems.

**FIGURE 2:**
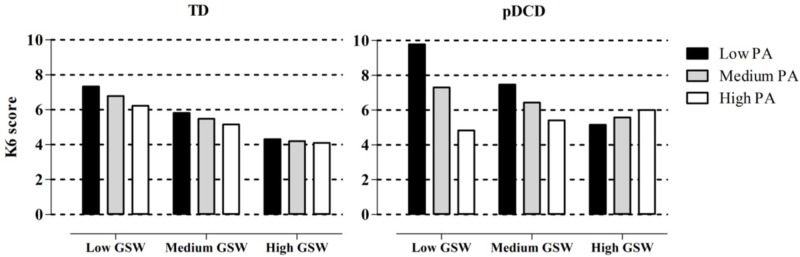
Internalizing problems in conditions with different levels of physical activity and global self-worth. TD, typically developing; pDCD, probable developmental coordination disorder; PA, physical activity; GSW, global self-worth; low, –1 SD; medium, mean; high, +1 SD.

In order to better understand the above relationships, we graphed the levels of internalizing problems at different levels of physical activity and global self-worth for both groups. Overall, as seen in **Figure 2**, levels of internalizing problems decreased with concurrent increases in physical activity and global self-worth in both TD children and children with pDCD. However, when compared to TD children, a greater reduction in internalizing problems was associated with increases in physical activity and global self-worth among children with pDCD.

## Discussion

To the best of our knowledge, this is the first study to examine the potential buffering (i.e., moderating) effects of physical activity and personal resources (i.e., global self-worth) on the relationship between motor impairments and internalizing problems based on the ESH (Cairney et al., 2013). Consistent with previous research, we found that children with pDCD had poorer motor coordination, lower levels of physical activity and global self-worth, and a greater amount of internalizing problems. Although the simple and additive moderation models were not significant, the significant moderated moderation of physical activity and global self-worth was found on the relationship between pDCD and internalizing problems. This suggests that the positive effect of physical activity on internalizing problems is variable across both groups (pDCD versus TD) and different levels of global self-worth. For example, among children with pDCD, low physical activity and low global self-worth is associated with higher levels of internalizing problems. However, there appears to be a protective effect of physical activity between children in these groups. Specifically, children with pDCD and low global self-worth but high physical activity show the lowest levels of internalizing problems. When considered together, both physical activity and global self-worth play important synergistic roles in predicting internalizing symptoms in both TD and pDCD children.

Previous studies have shown that participation in physical activity is associated with positive self-concept in youth (an indicator of self-worth), including physical self-concept, perceived competence, perceived appearance, and self-efficacy (Ryan and Dzewaltowski, 2002; Babic et al., 2014). However, this may not always be the case in children with motor impairments. Without appropriately and carefully selecting (and if necessary, adapting) physical activities, motor skills that are required by the activities may not match children’s actual motor abilities. As a result, motor difficulties that thwart participation in physical activities may conversely cause frustration or the sense of failure (Mandich et al., 2003; Rodger and Mandich, 2005), and further deteriorate mental health. This may also explain why this study found that there was only significant moderated moderation of physical activity and global self-worth on the relationship between pDCD and internalizing problems, highlighting the need for a comprehensive intervention integrating motor coordination training, participation in physical activity, and positive global self-worth in school-aged children with motor impairments. Hence, health professionals should bear in mind that children’s self-worth may also be developed through meaningful participation in physical activity where children not only practice motor skills, but also enhance their sense of mastery and self-esteem and perceptions of self-competence.

Although findings from the present study cannot directly ascertain causation (as in experimental or longitudinal designs), they provide important information pertaining intermediary variables (i.e., physical activity and global self-worth) that may be the target of future intervention. For instance, it is worth noting that conventional interventions or training programs for children with poor motor coordination or motor impairments are usually developed to address their motor difficulties (e.g., traditional physical or occupational therapy) and improve their performance on activities of daily living (e.g., Cognitive Orientation to Daily Occupational Performance or skill acquisition program; Smits-Engelsman et al., 2013). Even though the psychosocial health of children with lower levels of motor coordination may also be improved after conventional interventions or training programs, this is often collateral to the main goals of the therapy. To date, only a few interventions have targeted both motor and psychosocial components (e.g., *Animal Fun* or *Partnering for Changing*) to address overall well-being (Piek et al., 2010; Missiuna et al., 2012). Indeed, by collaborating with peers, parents, and school teachers, recent integrated programs have shown promise for positively affecting various psychosocial outcomes, such as prosocial behaviors, peer relations, and self-esteem (Piek et al., 2015; Cacola et al., 2016; Noordstar et al., 2017; Wilson and Harris, 2017). Nevertheless, there are controversial findings with regard to the effect of these motor skill intervention programs on internalizing problems which may be due to less emphasis on participation in physical activity in these programs (Piek et al., 2015; Cacola et al., 2016). Further inventions should be developed to simultaneously focus on motor skills, physical activity participation, and self-worth (or self-concept), which, to the best of our knowledge, no program has been specifically developed for children with DCD.

In addition to personal resources, such as global self-worth tested in this study, the ESH also identifies social resources, such as perceived social support, or individual resources such as social skills, as additional factors that may buffer the adverse effect of DCD on mental health and may play similar moderated moderation roles (Cairney et al., 2013). However, as neither of these constructs were available in the PHAST study, the present study was unable to include perceptions of social support from different sources (e.g., parents, classmates, or teachers) or individual resources (e.g., social skills), and thus, could not investigate these potential protective effects on internalizing problems in children with DCD. Nevertheless, it is worth noting that prior research has found that social skills and perceived social support mediate the relationship between motor difficulties and internalizing problems in children and adolescents (Wilson et al., 2013; Mancini et al., 2016b). This infers the potential moderation of social skills and perceived social support. As such, future research is needed to examine these variables, alongside the variables examined in the present study, to provide a more complete test of the ESH. As previously mentioned, this study was a cross-sectional data analysis which limits our ability to explore longitudinal changes in all variables and, specifically, our ability to infer causation between constructs. Although there are a few longitudinal studies that have been recently conducted investigating the mediating pathways addressed in the ESH, including the predictability of motor impairments for physical, psychosocial, and mental health later in life (Wagner et al., 2016; Harrowell et al., 2017), no study has investigated the moderating pathways tested in the present study. Thus, future research is needed to aid in the understanding of potential buffering mechanisms of internalizing problems in children with motor impairments, specifically DCD.

## Conclusion

In sum, this study highlights the importance of the coupling effect of both physical activity and global self-worth on mental health in children with pDCD. We encourage future researchers to develop interventions from a more holistic perspective and integrate physical aspects with psychological well-being. For example, in order to prevent or reduce internalizing problems (i.e., depressive or anxious symptoms) in school-aged children with motor impairments, health professionals and physical education teachers are encouraged to emphasize participation in physical activity and ways to enhance psychological support alongside motor skill training.

## Ethics Statement

All subjects gave written informed consent in accordance with the Declaration of Helsinki. The protocol was approved by Brock University.

## Author Contributions

Y-CL conceptualized and drafted the manuscript and also conducted the statistical analyses. JG contributed to the interpretation of the results and writing of the manuscript. JC was the main contributor of this study and responsible for the final approval of the manuscript.

## Conflict of Interest Statement

The authors declare that the research was conducted in the absence of any commercial or financial relationships that could be construed as a potential conflict of interest.
